# Reliability assessment of transformer insulating oil using accelerated life testing

**DOI:** 10.1038/s41598-022-26247-2

**Published:** 2022-12-15

**Authors:** Xingchun Wei, Zhiming Wang, Junfeng Guo

**Affiliations:** School of Mechanical and Electronic Engineering, Lanzhou University of Technology, Lanzhou, 730050 China

**Keywords:** Electrical and electronic engineering, Mechanical engineering

## Abstract

To improve the reliability and reduce the maintenance cost of transformer oil, a life prediction of transformer oil is needed so that the maintenance of transformer can be performed correctly. However, it is difficult to predict the reliable lifetime of transformer oil accurately because of its unkind operating condition at different environments. To solve this problem, based on the theory of accelerated life testing (ALT), a reliability assessment method for transformer insulating oil on a normal operational conditions is proposed. An inverse power Weibull distribution model for insulating oil lifetime with voltage is built. Numerical procedure of model parameter estimation is presented, the variances of model parameters and reliability indices, which including mean lifetime, reliability, reliable lifetime at given reliability and failure rate, are derived. The feasibility and correctness of the proposed method are validated by real lifetime data of transformer insulating oil in literature. The reliability of transformer insulating oil used at normal usage conditions are predicted by the proposed method, and the point and interval estimations of reliability indices are evaluated. The results show that reliable lifetime and mean lifetime under reliability limit should be considered simultaneously in repair or replacement of transformer insulating oil.

## Introduction

Transformer is a key equipment in electrical system transmission, it plays a very important role in stable operation of power system. If a failure occurs in transformer, it will bring huge losses to power enterprises and national economy. A lot of research data show that the main cause of transformer failure is the deterioration of insulation performance^1^. This is because that transformer is subjected to the interaction of thermal, electrical and mechanical stresses in operation conditions, and its insulation system is gradually aging and unable to meet the requirements of safe and reliable operation, which resulting in a failure at last^1^. The service life of oil-paper insulated transformer is determined by the life of insulating material which is mainly composed of insulating oil and solid insulation. Among them, the performance decline of transformer insulation oil is the main reason of transformer breakdown. On the other hand, it is found that voltage is the main factor which affecting the insulation performance of transformer oil. Therefore, at present, a lot of research work has been done on transformer insulation aging and insulation life prediction. Based on a single ageing test at higher temperature and the activation energy of the oxidation reaction obtained by non-iso-thermal Differential Scanning Calorimetry measurements. Dumitran et al.^2^ proposed a mineral oil lifetime estimation method. To prolong the service life of transformers, Chatterjee et al.^3^ developed predictive models for transformer health. Due to the uncertainty and complexity between transformer remaining life and its influence factors such as load and ambient temperature, AbU-Elanien and Salama^4^ applied a Monte Carlo method to estimate the thermal life of transformer insulation oil. Other commonly used methods for analyzing transformer insulation aging include neural network method^5–8^, fuzzy analysis method^9–12^, grey clustering method^13^, genetic algorithm^14,15^, deep learning^16,17^, support vector machine^18,19^ and hybrid methods^20–24^, etc.

However, due to the limitation of time and funds, it is impossible and unnecessary to carry out long-term life test of insulating materials according to actual operating conditions in engineering practice. In this case, an ideal solution is to use accelerated life testing (ALT) method. In other words, by an ALT of insulating materials under high stress level, fault data of transformer can be obtained in a short time, and the corresponding life model can be established, so as to extrapolate the life characteristics under normal operational conditions. ALT method can also provide a theoretical basis for transformer overhaul and maintenance plan, and then simultaneously determine the best time for transformer insulation oil overhaul and replacement^25–27^. In literature, the relationship between insulation oil voltage and lifetime is often described with inverse power law (IPL) model^28,29^, and failure times of insulation oil generally follow Weibull distribution^30–32^. Therefore, a Weibull IPL life model is applied in this paper to analyze the reliability and reliable life of transformer insulation oil.

Therefore, the novelty and main contributions of this work can be summarized as follows:

1. Numerical method of model parameter estimation of IPL-Weibull distribution for insulating oil lifetime is proposed, the variances of model parameters and reliability indices, which including mean lifetime, reliability, reliable lifetime and failure rate, are derived.
2. The reliability of transformer insulating oil used at usual operational conditions are predicted by the proposed method, and the point and interval estimations of reliability indices are evaluated. The feasibility and correctness of the proposed method are validated by real lifetime data of transformer insulating oil in literature.

The paper is organized as follows. In “Methodology”, the details on lifetime data modelling for transformer insulating oil using IPL-Weibull distribution, model parameter estimation, as well as the point and interval estimation for reliability indices are given. In “Case study”, some information about ALT for a real case in literature are described. Results and comparison with the existing method are presented with details in “Results and discussion”. Conclusions are drawn in the final and concluding section.

## Methodology

### Reliability modelling for transformer insulating oil with accelerated life testing

Some basic assumptions of ALT for insulating oil are given as follows:

1. The lifetime of insulating oil follows the Weibull distribution under usual use stress level and accelerated stress level;
2. The failure mechanism of insulating oil is the same at different stress levels;
3. The acceleration model of insulating oil is the same at each stress level.

Suppose that failure time *t* of insulating oil follows a two-parameter Weibull distribution, then the cumulative distribution function (CDF) *F*(*t*) and probability density function (pdf) *f*(*t*) of failure time *t* of insulating oil are respectively given by

1 $$F\left( t \right) = 1 - \exp \left[ { - \left( {{t \mathord{\left/ {\vphantom {t \eta }} \right. \kern-\nulldelimiterspace} \eta }} \right)^{\beta } } \right]$$

and

2 $$f\left( t \right) = \beta \eta^{ - \beta } t^{\beta - 1} \exp \left[ { - \left( {{t \mathord{\left/ {\vphantom {t \eta }} \right. \kern-\nulldelimiterspace} \eta }} \right)^{\beta } } \right]$$

where *η* is scale parameter and *β* is shape parameter, *η* > 0, *β* > 0.

The IPL model can be used for insulating oil where voltage is the main stress. Therefore, its characteristic life is

3 $$\eta \left( U \right) = {1 \mathord{\left/ {\vphantom {1 {\left( {KU^{N} } \right)}}} \right. \kern-\nulldelimiterspace} {\left( {KU^{N} } \right)}}$$

where *K* > 0, *N* > 0 are model parameters, and *U* > 0 is voltage stress.

Therefore, from Eqs. (1) and (3), the reliability *R* of insulating oil at given time *t* can be given by

4 $$R\left( t \right) = 1 - F\left( t \right) = \exp \left[ { - \left( {KU^{N} t} \right)^{\beta } } \right]$$

### Numerical method of model parameter estimation

Assume that *n* stress levels of insulating oil in ALT are *U*_1_ < *U*_2_ < … < *U*_*i*_ < … < *U*_*n*_ (*i* = 1, 2, …, *n*), its failure time at the *i*th stress level is *t*_*i*1_ < *t*_*i*2_ < … < *t*_*ij*_ < … < $t_{{im_{i} }}$(*j* = 1, 2, …, *m*_*i*_), then substituting Eq. (3) into Eq. (2), the responding pdf of all failure time of insulating oil in ALT is

5 $$f\left( {t_{i,j} } \right) = \beta \left( {KU_{i}^{N} } \right)^{\beta } t_{i,j}^{\beta - 1} \exp \left[ { - \left( {KU_{{_{i} }}^{N} t_{i,j} } \right)^{\beta } } \right]$$

According Eq. (5), the likelihood function of failure time *t*_*i,j*_ of insulating oil is

6 $$L\left( {t_{i,j} ;\beta ,N,K} \right) = \prod\limits_{i = 1}^{n} {\left( {\prod\limits_{j = 1}^{{m_{i} }} {f\left( {t_{i,j} } \right)} } \right)} = \prod\limits_{i = 1}^{n} {\prod\limits_{j = 1}^{{m_{i} }} {\left[ {\beta \left( {KU_{i}^{N} } \right)^{\beta } t_{i,j}^{\beta - 1} \exp \left( { - \left( {KU_{i}^{N} t_{i,j} } \right)^{\beta } } \right)} \right]} }$$

Thus, the corresponding log-likelihood function is

7 $$\Lambda = \ln L\left( {t_{i,j} ;\beta ,N,K} \right) = \sum\limits_{i = 1}^{n} {\sum\limits_{j = 1}^{{m_{i} }} {\ln \left[ {\beta \left( {KU_{i}^{N} } \right)^{\beta } t_{i,j}^{\beta - 1} } \right]} } - \sum\limits_{i = 1}^{n} {\sum\limits_{k = 1}^{{m_{i} }} {\left( {KU_{i}^{N} t_{i,j} } \right)^{\beta } } }$$

Taking the first partial derivatives of Eq. (7) with respect to the model parameters *β*, *K* and *N*, and setting them equal to zero, respectively. The maximum likelihood estimates (MLE) of model parameters satisfy Eqs. (8)–(10):

8 $$\frac{\partial \Lambda }{{\partial \beta }} = \sum\limits_{i = 1}^{n} {\sum\limits_{j = 1}^{{m_{i} }} {\left[ {\frac{1}{\beta } + \ln (KU_{i}^{N} t_{i,j} )} \right]} } - \sum\limits_{i = 1}^{n} {\sum\limits_{j = 1}^{{m_{i} }} {\left[ {\left( {KU_{i}^{N} t_{i,j} } \right)^{\beta } \ln (KU_{i}^{N} t_{i,j} )} \right] = 0} }$$

9 $$\frac{\partial \Lambda }{{\partial N}} = \beta \sum\limits_{i = 1}^{n} {\sum\limits_{j = 1}^{{m_{i} }} {\left\{ {\ln U_{i} \left[ {1 - \left( {KU_{i}^{N} t_{i,j} } \right)^{\beta } } \right]} \right\}} = 0}$$

10 $$\frac{\partial \Lambda }{{\partial K}} = \frac{\beta }{K}\sum\limits_{i = 1}^{n} {\sum\limits_{j = 1}^{{m_{i} }} {\left[ {1 - \left( {KU_{i}^{N} t_{i,j} } \right)^{\beta } } \right] = 0} }$$

However, Eqs. (8)-(10) have no closed-form solutions, and a numerical method, which consists of three steps, is needed.

Step 1: Estimate the first model parameter *β* using the least square method. Twice taking the logarithmic of Eq. (1) with some mathematical transformations, the following Eq. (11) can be obtained as
  11 $$\ln \left[ { - \ln \left( {1 - F_{i} \left( {t_{i,j} } \right)} \right)} \right] = \beta_{i} \left( {\ln t_{i,j} - \ln \eta_{i} } \right)$$
  where *F*_*i*_ (*t*_*i, j*_) can be given by midpoint estimate or median rank estimate, and the estimators are
  12 $$\hat{F}_{i} (t_{i,j} ) = {{\left( {j - 0.5} \right)} \mathord{\left/ {\vphantom {{\left( {j - 0.5} \right)} {m_{i} }}} \right. \kern-\nulldelimiterspace} {m_{i} }},j = 1,2,...,m_{i}$$
  and
  13 $$\hat{F}_{i} (t_{i,j} ) = {{\left( {j - 0.3} \right)} \mathord{\left/ {\vphantom {{\left( {j - 0.3} \right)} {\left( {m_{i} + 0.4} \right)}}} \right. \kern-\nulldelimiterspace} {\left( {m_{i} + 0.4} \right)}},j = 1,2,...,m_{i}$$

Therefore, the least square method^33^ can be used to obtain an estimate of the parameter *β*_*i*_ by combining Eq. (11) with Eqs. (12) or (13). ALT does not change failure mechanism, and thus the estimators of *β*_*i*_ (*i* = 1, 2, …, *n*) should be equal each other. However, there exit some errors and uncertainty in test, the estimators of *β*_*i*_ are not exactly the same. Therefore, its weighted estimated value can be given by

14 $$\hat{\beta } = {{\sum\limits_{i = 1}^{n} {m_{i} } \hat{\beta }_{i} } \mathord{\left/ {\vphantom {{\sum\limits_{i = 1}^{n} {m_{i} } \hat{\beta }_{i} } {\sum\limits_{i = 1}^{n} {m_{i} } }}} \right. \kern-\nulldelimiterspace} {\sum\limits_{i = 1}^{n} {m_{i} } }}$$

Step 2: Estimate the second model parameter *K* with an iterative method. After some mathematical calculations, Eq. (9) and Eq. (10) can be changed as
  15 $$\sum\limits_{i = 1}^{n} {m_{i} \ln U_{i} } = K^{\beta } \sum\limits_{i = 1}^{n} {\sum\limits_{j = 1}^{{m_{i} }} {\left( {U_{i}^{N} t_{i,j} } \right)^{\beta } \ln U_{i} } }$$
  16 $$\sum\limits_{i = 1}^{n} {m_{i} } = K^{\beta } \sum\limits_{i = 1}^{n} {\sum\limits_{j = 1}^{{m_{i} }} {\left( {U_{i}^{N} t_{i,j} } \right)^{\beta } } }$$

Divided the left and right sides of Eq. (16) by the corresponding two sides of Eq. (15), one can get

17 $$\frac{{\sum\limits_{i = 1}^{n} {m_{i} } }}{{\sum\limits_{i = 1}^{n} {\left( {m_{i} \ln U_{i} } \right)} }} = \frac{{\sum\limits_{i = 1}^{n} {\sum\limits_{j = 1}^{{m_{i} }} {\left( {U_{i}^{N} t_{i,j} } \right)^{{\hat{\beta }}} } } }}{{\sum\limits_{i = 1}^{n} {\sum\limits_{j = 1}^{{m_{i} }} {\left[ {\left( {U_{i}^{N} t_{i,j} } \right)^{{\hat{\beta }}} \ln U_{i} } \right]} } }}$$

Equation (17) contains only parameter *N*, and its estimated value $\hat{N}$ can be solved by an iterative method. One iterative expression for the second parameter *N* would be

18 $$\hat{N}^{{\left( {l + 1} \right)}} = \frac{1}{{\ln U_{1} }}\left\{ {\ln \left[ {D - \sum\limits_{j = 2}^{{m_{i} }} {\left( {U_{1}^{{\hat{N}^{\left( l \right)} }} t_{1,j} } \right)^{{\hat{\beta }}} } - \sum\limits_{i = 2}^{n} {\sum\limits_{j = 1}^{{m_{i} }} {\left( {U_{i}^{{\hat{N}^{\left( l \right)} }} t_{i,j} } \right)^{{\hat{\beta }}} } } } \right]^{{{1 \mathord{\left/ {\vphantom {1 {\hat{\beta }}}} \right. \kern-\nulldelimiterspace} {\hat{\beta }}}}} - \ln t_{1,1} } \right\}$$

where $\hat{N}^{{\left( {l + 1} \right)}}$ and $\hat{N}^{\left( l \right)}$ are the (*l* + 1)th and *l* th iteration values of parameter *N*, and

$$D = {{\left( {\sum\limits_{i = 1}^{n} {m_{i} } } \right) \times \left( {\sum\limits_{i = 1}^{n} {\sum\limits_{j = 1}^{{m_{i} }} {\left[ {\left( {U_{i}^{{\hat{N}^{\left( l \right)} }} t_{i,j} } \right)^{{\hat{\beta }}} \ln U_{i} } \right]} } } \right)} \mathord{\left/ {\vphantom {{\left( {\sum\limits_{i = 1}^{n} {m_{i} } } \right) \times \left( {\sum\limits_{i = 1}^{n} {\sum\limits_{j = 1}^{{m_{i} }} {\left[ {\left( {U_{i}^{{\hat{N}^{\left( l \right)} }} t_{i,j} } \right)^{{\hat{\beta }}} \ln U_{i} } \right]} } } \right)} {\sum\limits_{i = 1}^{n} {\left( {m_{i} \ln U_{i} } \right)} }}} \right. \kern-\nulldelimiterspace} {\sum\limits_{i = 1}^{n} {\left( {m_{i} \ln U_{i} } \right)} }}$$

Step 3: Estimate the last model parameter *N* by the iterative method. Finally, substituting the estimated value $\hat{N}$ into Eq. (8), the estimated value $\hat{K}$ of the last parameter *K* can be obtain, and its (*r* + 1) th iteration value is
  19 $$\hat{K}_{{^{1} }}^{{^{{\left( {r + 1} \right)}} }} = \left( {\frac{{\sum\limits_{i = 1}^{n} {\left( {{{m_{i} } \mathord{\left/ {\vphantom {{m_{i} } {\hat{\beta }}}} \right. \kern-\nulldelimiterspace} {\hat{\beta }}}} \right)} + \sum\limits_{i = 1}^{n} {\sum\limits_{j = 1}^{{m_{i} }} {\ln \left( {\hat{K}_{{^{1} }}^{{^{\left( r \right)} }} U_{i}^{{\hat{N}}} t_{i,j} } \right)} } }}{{\sum\limits_{i = 1}^{n} {\sum\limits_{j = 1}^{{m_{i} }} {\left[ {\left( {U_{i}^{{\hat{N}}} t_{i,j} } \right)^{{\hat{\beta }}} \ln \left( {\hat{K}_{{^{1} }}^{{^{\left( r \right)} }} U_{i}^{{\hat{N}}} t_{i,j} } \right)} \right]} } }}} \right)^{{{1 \mathord{\left/ {\vphantom {1 {\hat{\beta }}}} \right. \kern-\nulldelimiterspace} {\hat{\beta }}}}}$$
  where $\hat{K}_{1}^{\left( r \right)}$ is the *r* th iteration value of parameter *K*. When the estimated value $\hat{K}$ of parameter *K* is obtained by using Eq. (9) or Eq. (10), its value is
  20 $$\hat{K}_{2} = \left( {{{\sum\limits_{i = 1}^{n} {\left( {m_{i} \ln U_{i} } \right)} } \mathord{\left/ {\vphantom {{\sum\limits_{i = 1}^{n} {\left( {m_{i} \ln U_{i} } \right)} } {\sum\limits_{i = 1}^{n} {\sum\limits_{j = 1}^{{m_{i} }} {\left[ {\left( {U_{i}^{{\hat{N}}} t_{i,j} } \right)^{{\hat{\beta }}} \ln U_{i} } \right]} } }}} \right. \kern-\nulldelimiterspace} {\sum\limits_{i = 1}^{n} {\sum\limits_{j = 1}^{{m_{i} }} {\left[ {\left( {U_{i}^{{\hat{N}}} t_{i,j} } \right)^{{\hat{\beta }}} \ln U_{i} } \right]} } }}} \right)^{{{1 \mathord{\left/ {\vphantom {1 {\hat{\beta }}}} \right. \kern-\nulldelimiterspace} {\hat{\beta }}}}}$$
  or
  21 $$\hat{K}_{3} = \left( {{{\sum\limits_{i = 1}^{n} {m_{i} } } \mathord{\left/ {\vphantom {{\sum\limits_{i = 1}^{n} {m_{i} } } {\sum\limits_{i = 1}^{n} {\sum\limits_{j = 1}^{{m_{i} }} {\left( {U_{i}^{{\hat{N}}} t_{i,j} } \right)^{{\hat{\beta }}} } } }}} \right. \kern-\nulldelimiterspace} {\sum\limits_{i = 1}^{n} {\sum\limits_{j = 1}^{{m_{i} }} {\left( {U_{i}^{{\hat{N}}} t_{i,j} } \right)^{{\hat{\beta }}} } } }}} \right)^{{{1 \mathord{\left/ {\vphantom {1 {\hat{\beta }}}} \right. \kern-\nulldelimiterspace} {\hat{\beta }}}}}$$

In general, at this case, $\hat{K}_{1} \ne \hat{K}_{2} = \hat{K}_{3}$, the estimated value $\hat{K}$ can be selected according to the maximum log-likelihood value of Eq. (7), and given by

22 $$\hat{K} = \left\{ \begin{gathered} \hat{K}_{1} {, }\ln L\left( {\hat{\beta },\hat{N},\hat{K}_{1} } \right) \ge \ln L\left( {\hat{\beta },\hat{N},\hat{K}_{2} } \right) \hfill \\ \hat{K}_{2} {, }\ln L\left( {\hat{\beta },\hat{N},\hat{K}_{1} } \right) < \ln L\left( {\hat{\beta },\hat{N},\hat{K}_{2} } \right) \hfill \\ \end{gathered} \right.$$

Thus, all estimated values of three model parameters can be obtained.

### Interval estimates for model parameters and reliability indices

To estimate the confidence bounds of model parameters and reliability indicators, their variances are needed. The variance–covariance matrix of model parameters can be given by Fisher information matrix method as follows^34^:

23 $$\left[ {\begin{array}{*{20}c} {{\text{Var}}(\hat{\beta })} & {{\text{Cov}}(\hat{\beta },\hat{N})} & {{\text{Cov}}(\hat{\beta },\hat{K})} \\ {{\text{Cov}}(\hat{N},\hat{\beta })} & {{\text{Var}}(\hat{N})} & {{\text{Cov}}(\hat{N},\hat{K})} \\ {{\text{Cov}}(\hat{K},\hat{\beta })} & {{\text{Cov}}(\hat{K},\hat{N})} & {{\text{Var}}(\hat{K})} \\ \end{array} } \right] = \left[ {\begin{array}{*{20}c} {\Delta_{11} } & {\Delta_{12} } & {\Delta_{13} } \\ {\Delta_{21} } & {\Delta_{22} } & {\Delta_{23} } \\ {\Delta_{31} } & {\Delta_{32} } & {\Delta_{33} } \\ \end{array} } \right]_{{\beta = \hat{\beta }, \, N = \hat{N}, \, K = \hat{K}}}^{ - 1}$$

where

$$\begin{gathered} \Delta_{11} = - \frac{{\partial^{2} \Lambda }}{{\partial \beta^{2} }} = \sum\limits_{i = 1}^{n} {\frac{{m_{i} }}{{\beta^{2} }} + } \sum\limits_{i = 1}^{n} {\sum\limits_{j = 1}^{{m_{i} }} {\left[ {\left( {KU_{i}^{N} t_{i,j} } \right)^{\beta } \ln^{2} \left( {KU_{i}^{N} t_{i,j} } \right)} \right]} } \hfill \\ \Delta_{22} = - \frac{{\partial^{2} \Lambda }}{{\partial N^{2} }} = \beta^{2} \sum\limits_{i = 1}^{n} {\sum\limits_{j = 1}^{{m_{i} }} {\left[ {\left( {KU_{i}^{N} t_{i,j} } \right)^{\beta } \ln^{2} U_{i} } \right]} } \hfill \\ \Delta_{33} = - \frac{{\partial^{2} \Lambda }}{{\partial K^{2} }} = \frac{\beta }{{K^{2} }}\left[ {\sum\limits_{i = 1}^{n} {m_{i} + \left( {\beta - 1} \right)\sum\limits_{i = 1}^{n} {\sum\limits_{j = 1}^{{m_{i} }} {\left( {KU_{i}^{N} t_{i,j} } \right)^{\beta } } } } } \right] \hfill \\ \end{gathered}$$

24 $$\begin{gathered} \Delta_{12} = \Delta_{21} = - \frac{{\partial^{2} \Lambda }}{\partial \beta \partial N} = \sum\limits_{i = 1}^{n} {\sum\limits_{j = 1}^{{m_{i} }} {\ln U_{i} \left\{ {\left( {KU_{i}^{N} t_{i,j} } \right)^{\beta } \left[ {\beta \ln (KU_{i}^{N} t_{i,j} ) + 1} \right]} \right\}} } - \sum\limits_{i = 1}^{n} {m_{i} \ln U_{i} } \hfill \\ \Delta_{13} = \Delta_{31} = - \frac{{\partial^{2} \Lambda }}{\partial \beta \partial K} = \frac{1}{K}\sum\limits_{i = 1}^{n} {\sum\limits_{j = 1}^{{m_{i} }} {\left\{ {\left( {KU_{i}^{N} t_{i,j} } \right)^{\beta } \left[ {\beta \ln (KU_{i}^{N} t_{i,j} ) + 1} \right]} \right\}} } - \frac{1}{K}\sum\limits_{i = 1}^{n} {m_{i} } \hfill \\ \Delta_{23} = \Delta_{32} = - \frac{{\partial^{2} \Lambda }}{\partial N\partial K} = \frac{{\beta^{2} }}{K}\sum\limits_{i = 1}^{n} {\sum\limits_{j = 1}^{{m_{i} }} {\left[ {\left( {KU_{i}^{N} t_{i,j} } \right)^{\beta } \ln U_{i} } \right]} } \hfill \\ \end{gathered}$$

In general, the MLE of model parameters are asymptotically a normal distribution. Therefore, the interval estimation IE_***θ***_ of the model parameter ***θ*** = (*β*, *N*, *K*) with a confidence of 100(1-*α*)% can be constructed as

25 $${\text{IE}}_{{\varvec{\theta}}} = \hat{\user2{\theta }} \pm z_{\alpha /2} \sqrt {{\text{Var}}\left( {\hat{\user2{\theta }}} \right)} = \left[ {\hat{\user2{\theta }} - z_{\alpha /2} \sqrt {{\text{Var}}\left( {\hat{\user2{\theta }}} \right)} , \, \hat{\user2{\theta }} + z_{\alpha /2} \sqrt {{\text{Var}}\left( {\hat{\user2{\theta }}} \right)} } \right] = \left[ {{\varvec{\theta}}_{L} ,{\varvec{\theta}}_{U} } \right]$$

where $\hat{\user2{\theta }}$ is the estimated value of model parameter,${\text{Var}}\left( {\hat{\user2{\theta }}} \right)$ is the variance of model parameter, *Z*_*α*/2_ is the quantile of standard normal distribution, *L* and *U* are the lower bound and upper bound, respectively.

When model parameters are non-negative, they are more close to follow a lognormal distribution^34^. Therefore,

$${{\left( {\ln \hat{\user2{\theta }} - \ln {\varvec{\theta}}} \right)} \mathord{\left/ {\vphantom {{\left( {\ln \hat{\user2{\theta }} - \ln {\varvec{\theta}}} \right)} {\sqrt {{\text{Var}}\left( {\hat{\user2{\theta }}} \right)} }}} \right. \kern-\nulldelimiterspace} {\sqrt {{\text{Var}}\left( {\hat{\user2{\theta }}} \right)} }} \sim N\left( {0,1} \right)$$

Thus, the log-transformed interval estimation IE_ln***θ***_ of model parameter ***θ*** can be given as

26 $$\begin{aligned} {\text{IE}}_{{\ln {\varvec{\theta}}}} & = \ln \left( {\hat{\user2{\theta }}} \right) \pm z_{\alpha /2} \sqrt {{\text{Var}}\left( {\ln \left( {\hat{\user2{\theta }}} \right)} \right)} = \ln \left( {\hat{\user2{\theta }}} \right) \pm z_{\alpha /2} \sqrt {\left( {\frac{1}{{\hat{\user2{\theta }}}}} \right)^{2} {\text{Var}}\left( {\hat{\user2{\theta }}} \right)} \\ & = \ln \left( {\hat{\user2{\theta }}} \right) \pm {{z_{\alpha /2} \sqrt {{\text{Var}}\left( {\hat{\user2{\theta }}} \right)} } \mathord{\left/ {\vphantom {{z_{\alpha /2} \sqrt {{\text{Var}}\left( {\hat{\user2{\theta }}} \right)} } {\hat{\user2{\theta }}}}} \right. \kern-\nulldelimiterspace} {\hat{\user2{\theta }}}} \\ \end{aligned}$$

So, from Eq. (26), the interval estimation IE_***θ***_ of model parameter ***θ*** is

27 $$\begin{aligned} {\text{IE}}_{{\varvec{\theta}}} & = \hat{\user2{\theta }}\exp \left( { \pm {{z_{\alpha /2} \sqrt {{\text{Var}}\left( {\hat{\user2{\theta }}} \right)} } \mathord{\left/ {\vphantom {{z_{\alpha /2} \sqrt {{\text{Var}}\left( {\hat{\user2{\theta }}} \right)} } {\hat{\user2{\theta }}}}} \right. \kern-\nulldelimiterspace} {\hat{\user2{\theta }}}}} \right) \\ & = \left[ {\hat{\user2{\theta }}\exp \left( { - {{z_{\alpha /2} \sqrt {{\text{Var}}\left( {\hat{\user2{\theta }}} \right)} } \mathord{\left/ {\vphantom {{z_{\alpha /2} \sqrt {{\text{Var}}\left( {\hat{\user2{\theta }}} \right)} } {\hat{\user2{\theta }}}}} \right. \kern-\nulldelimiterspace} {\hat{\user2{\theta }}}}} \right), \, \hat{\user2{\theta }}\exp \left( {{{z_{\alpha /2} \sqrt {{\text{Var}}\left( {\hat{\user2{\theta }}} \right)} } \mathord{\left/ {\vphantom {{z_{\alpha /2} \sqrt {{\text{Var}}\left( {\hat{\user2{\theta }}} \right)} } {\hat{\user2{\theta }}}}} \right. \kern-\nulldelimiterspace} {\hat{\user2{\theta }}}}} \right)} \right] \\ \end{aligned}$$

Commonly used reliability indices include mean time-to-failure *t*_MTTF_, the reliability *R* with a given time *t*, the reliable life *t*_*R*_ for a given reliability and failure rate function.

Based on Eqs. (2) and (4), *t*_MTTF_ can be obtained as follows:

28 $$t_{{{\text{MTTF}}}} = E\left( t \right) = \int_{0}^{\infty } t f\left( t \right){\text{d}}t = \int_{0}^{\infty } {R\left( t \right)} {\text{d}}t = \frac{1}{{KU^{N} }}\Gamma \left( {1 + \left( {{1 \mathord{\left/ {\vphantom {1 \beta }} \right. \kern-\nulldelimiterspace} \beta }} \right)} \right)$$

and the variance of *t*_MTTF_ is

29 $$\begin{aligned} {\text{Var}}\left( {t_{{{\text{MTTF}}}} } \right) & = \int_{0}^{\infty } {\left[ {t - E\left( t \right)} \right]} f\left( t \right){\text{d}}t = \int_{0}^{\infty } {t^{2} } f\left( t \right){\text{d}}t - \left[ {E\left( t \right)} \right]^{2} \\ & = \frac{1}{{K^{2} U^{2N} }}\left[ {\Gamma \left( {1 + {2 \mathord{\left/ {\vphantom {2 \beta }} \right. \kern-\nulldelimiterspace} \beta }} \right) - \Gamma^{2} \left( {1 + {1 \mathord{\left/ {\vphantom {1 \beta }} \right. \kern-\nulldelimiterspace} \beta }} \right)} \right] \\ \end{aligned}$$

where Г(·) is gamma function, and according to Eq. (4), the reliable life *t*_*R*_ for a specified reliability can be given by

30 $$t\left( R \right) = {{\left( { - \ln R} \right)^{{{1 \mathord{\left/ {\vphantom {1 \beta }} \right. \kern-\nulldelimiterspace} \beta }}} } \mathord{\left/ {\vphantom {{\left( { - \ln R} \right)^{{{1 \mathord{\left/ {\vphantom {1 \beta }} \right. \kern-\nulldelimiterspace} \beta }}} } {\left( {KU^{N} } \right)}}} \right. \kern-\nulldelimiterspace} {\left( {KU^{N} } \right)}}$$

According to Eqs. (2) and (4), the failure rate function of insulating oil is got by

31 $$\lambda \left( t \right) = {{f\left( t \right)} \mathord{\left/ {\vphantom {{f\left( t \right)} {R\left( t \right)}}} \right. \kern-\nulldelimiterspace} {R\left( t \right)}} = \beta \left( {KU^{N} } \right)^{\beta } t^{\beta - 1}$$

To obtain the confidence bound of reliability, reliable life and failure rate of insulating oil, the equivalent change method is applied. In Eq. (4), set $u = \ln \left[ {\left( {KU^{N} t} \right)^{\beta } } \right] = \beta \left( {\ln K + N\ln U + \ln t} \right)$, then

32 $$\begin{aligned} {\text{Var}}\left( {\hat{u}} \right) & = \left( {\frac{\partial u}{{\partial \beta }}} \right)^{2} {\text{Var}}\left( {\hat{\beta }} \right) + \left( {\frac{\partial u}{{\partial N}}} \right)^{2} {\text{Var}}\left( {\hat{N}} \right) + \left( {\frac{\partial u}{{\partial K}}} \right)^{2} {\text{Var}}\left( {\hat{K}} \right) \\ & \;\;\;{ + }2\left( {\frac{\partial u}{{\partial \beta }}} \right)\left( {\frac{\partial u}{{\partial N}}} \right){\text{Cov}}\left( {\hat{\beta },\hat{N}} \right) + 2\left( {\frac{\partial u}{{\partial \beta }}} \right)\left( {\frac{\partial u}{{\partial K}}} \right){\text{Cov}}\left( {\hat{\beta },\hat{K}} \right) + 2\left( {\frac{\partial u}{{\partial N}}} \right)\left( {\frac{\partial u}{{\partial K}}} \right){\text{Cov}}\left( {\hat{N},\hat{K}} \right) \\ & = \left( {{u \mathord{\left/ {\vphantom {u \beta }} \right. \kern-\nulldelimiterspace} \beta }} \right)^{2} {\text{Var}}\left( {\hat{\beta }} \right) + \left( {\beta \ln U} \right)^{2} {\text{Var}}\left( {\hat{N}} \right) + \left( {{\beta \mathord{\left/ {\vphantom {\beta K}} \right. \kern-\nulldelimiterspace} K}} \right)^{2} {\text{Var}}\left( {\hat{K}} \right) \\ & \;\;\;{ + }\left( {2u\ln U} \right){\text{Cov}}\left( {\hat{\beta },\hat{N}} \right) + \left( {{{2u} \mathord{\left/ {\vphantom {{2u} K}} \right. \kern-\nulldelimiterspace} K}} \right){\text{Cov}}\left( {\hat{\beta },\hat{K}} \right) + \left( {{{\left( {2\beta^{2} \ln U} \right)} \mathord{\left/ {\vphantom {{\left( {2\beta^{2} \ln U} \right)} {\hat{K}}}} \right. \kern-\nulldelimiterspace} {\hat{K}}}} \right){\text{Cov}}\left( {\hat{N},\hat{K}} \right) \\ \end{aligned}$$

So, after obtaining the lower *u*_*L*_ and upper bound *u*_*U*_ of *u* with Eqs. (25), (27) and (32), the interval estimation of reliability *R* can be given by

33 $${\text{IE}}_{R} = \left[ {\exp \left( { - \exp u_{U} } \right), \, \exp \left( { - \exp u_{L} } \right)} \right]$$

Similarly, in Eq. (30), set $v = \ln t = \frac{1}{\beta }\ln \left( { - \ln R} \right) - \ln K - N\ln U$, then

34 $$\begin{aligned} {\text{Var}}\left( {\hat{v}} \right) & = \left[ {{{\ln \left( { - \ln R} \right)} \mathord{\left/ {\vphantom {{\ln \left( { - \ln R} \right)} {\hat{\beta }^{2} }}} \right. \kern-\nulldelimiterspace} {\hat{\beta }^{2} }}} \right]^{2} {\text{Var}}\left( {\hat{\beta }} \right) + \left( {\ln^{2} U} \right){\text{Var}}\left( {\hat{N}} \right) + \left( {{1 \mathord{\left/ {\vphantom {1 {\hat{K}}}} \right. \kern-\nulldelimiterspace} {\hat{K}}}} \right)^{2} {\text{Var}}\left( {\hat{K}} \right) \\ & \;\;\;{ + }\left[ {{{2\ln U\ln \left( { - \ln R} \right)} \mathord{\left/ {\vphantom {{2\ln U\ln \left( { - \ln R} \right)} {\hat{\beta }^{2} }}} \right. \kern-\nulldelimiterspace} {\hat{\beta }^{2} }}} \right]{\text{Cov}}\left( {\hat{\beta },\hat{N}} \right) + \left[ {{{2\ln \left( { - \ln R} \right)} \mathord{\left/ {\vphantom {{2\ln \left( { - \ln R} \right)} {\left( {\hat{K}\hat{\beta }^{2} } \right)}}} \right. \kern-\nulldelimiterspace} {\left( {\hat{K}\hat{\beta }^{2} } \right)}}} \right]{\text{Cov}}\left( {\hat{\beta },\hat{K}} \right) \\ & \;\;\;{ + }\left( {{{2\ln U} \mathord{\left/ {\vphantom {{2\ln U} {\hat{K}}}} \right. \kern-\nulldelimiterspace} {\hat{K}}}} \right){\text{Cov}}\left( {\hat{N},\hat{K}} \right) \\ \end{aligned}$$

The confidence interval [*v*_*L*_, *v*_*P*_] of *v* can also be estimated using Eqs. (25), (27) and (34). Finally, the interval estimate of reliable life is given as

35 $${\text{IE}}_{t} = \left[ {\exp v_{L} , \, \exp v_{U} } \right]$$

Finally, in Eq. (31), set $w = \ln \lambda = \ln \beta + \beta \left( {\ln K + N\ln U} \right) + \left( {\beta - 1} \right)\ln t$, then

36 $$\begin{aligned} {\text{Var}}\left( {\hat{w}} \right) & = \left[ {\left( {{1 \mathord{\left/ {\vphantom {1 {\hat{\beta }}}} \right. \kern-\nulldelimiterspace} {\hat{\beta }}}} \right) + \ln \left( {\hat{K}U^{{\hat{N}}} t} \right)} \right]^{2} {\text{Var}}\left( {\hat{\beta }} \right) + \left( {\hat{\beta }\ln U} \right)^{2} {\text{Var}}\left( {\hat{N}} \right) + \left( {{{\hat{\beta }} \mathord{\left/ {\vphantom {{\hat{\beta }} {\hat{K}}}} \right. \kern-\nulldelimiterspace} {\hat{K}}}} \right)^{2} {\text{Var}}\left( {\hat{K}} \right) \\ & \;\;\;{ + }2\ln U\left[ {1 + \hat{\beta }\ln \left( {\hat{K}U^{{\hat{N}}} t} \right)} \right]{\text{Cov}}\left( {\hat{\beta },\hat{N}} \right) + \frac{2}{{\hat{K}}}\left[ {1 + \hat{\beta }\ln \left( {\hat{K}U^{{\hat{N}}} t} \right)} \right]{\text{Cov}}\left( {\hat{\beta },\hat{K}} \right) \\ & \;\;\;{ + }\left( {{{2\hat{\beta }^{2} \ln U} \mathord{\left/ {\vphantom {{2\hat{\beta }^{2} \ln U} {\hat{K}}}} \right. \kern-\nulldelimiterspace} {\hat{K}}}} \right){\text{Cov}}\left( {\hat{N},\hat{K}} \right) \\ \end{aligned}$$

The interval estimate of failure rate can also be given as

37 $${\text{IE}}_{\lambda } = \left[ {\exp w_{L} , \, \exp w_{U} } \right]$$

## Case study

Using constant ALT, Ref.^35^ gives 76 failure-to-time data of a transformer insulation oil under *n* = 7 high voltage stress levels including *U*_1_ = 26, *U*_2_ = 28, *U*_3_ = 30, *U*_4_ = 32, *U*_5_ = 34, *U*_6_ = 36 and *U*_7_ = 38 kV, the numbers of failure time data for each stress level are *m*_1_ = 3, *m*_2_ = 5, *m*_3_ = 11, *m*_4_ = 15, *m*_5_ = 19, *m*_6_ = 15 and *m*_7_ = 8, respectively, the usual use voltage is 20 kV. The testing data are shown in Table 1.

**Table 1:** Constant ALT data for a transformer insulating oil with different voltages.

Stress level *i*	Voltage stress (*U*_*i*_/kV)	Failure times (*t*_*i,j*_/min)
1	26	5.79, 1579.52, 2323.70
2	28	68.85, 108.29, 110.29, 426.07, 1067.60
3	30	7.74, 17.05, 20.46, 21.02, 22.66, 43.40, 47.30, 139.07, 144.12, 175.88, 194.90
4	32	0.27, 0.40, 0.69, 0.79, 2.75, 3.91, 9.88, 13.95, 15.93, 27.80, 53.24, 82.85, 89.29, 100.58, 215.10
5	34	0.19, 0.78, 0.96, 1.31, 2.78, 3.16, 4.15, 4.67, 4.85, 6.50, 7.35, 8.01, 8.27, 12.06, 31.75, 32.52, 33.91, 36.71, 72.89
6	36	0.35, 0.59, 0.96, 0.99, 1.69, 1.97, 2.07, 2.58, 2.71, 2.90, 3.67, 3.99, 5.35, 13.77, 25.50
7	38	0.09, 0.39, 0.47, 0.73, 0.74, 1.13, 1.40, 2.38

## Results and discussion

When the least square method is used to estimate shape parameter *β* of accelerated life model, the midpoint estimate and median rank estimate can be used to fit the reliability of insulating oil. The estimates $\hat{\beta }$ of shape parameter *β* are shown in Table 2. It can be seen that the different estimate methods give different results. At the same time, even the same method is used, the estimated results for shape parameter *β* in the different stress level *i* are also different.

**Table 2:** The least square estimate for shape parameter.

Method	Shape parameter estimators							
	$\hat{\beta }_{1}$	$\hat{\beta }_{2}$	$\hat{\beta }_{3}$	$\hat{\beta }_{4}$	$\hat{\beta }_{5}$	$\hat{\beta }_{6}$	$\hat{\beta }_{7}$	$\hat{\beta }$
Midpoint estimate	0.3191	0.9293	1.0373	0.5425	0.8018	1.0594	1.2030	0.8671
Median rank estimate	0.2670	0.8212	0.9587	0.5090	0.7550	0.9898	1.0812	0.8017

**Figure 1:**
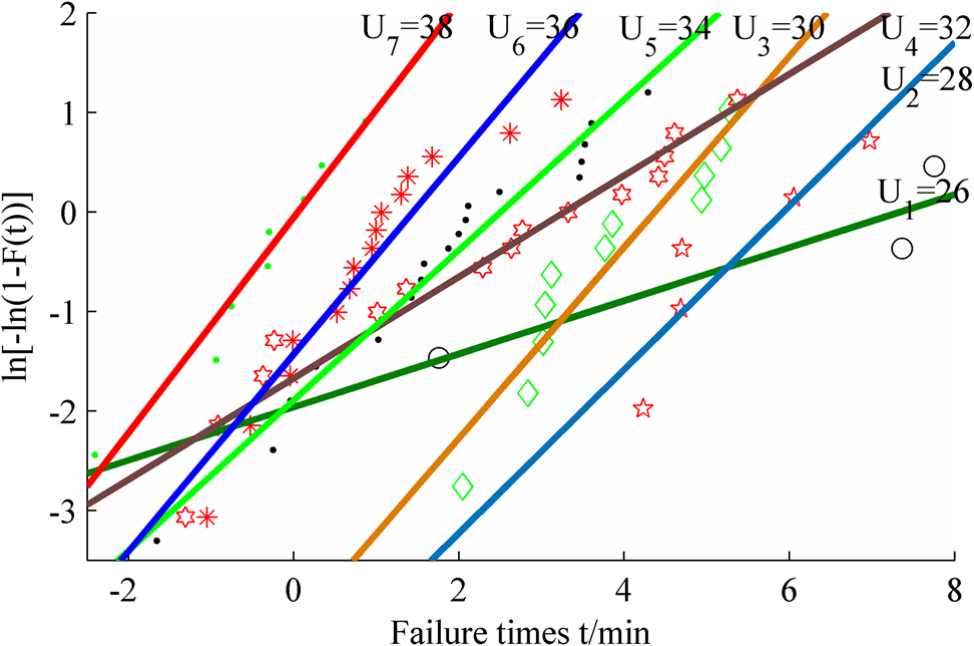
Probability plot under different voltages for insulating oil. Figure created using Matlab R2014a (https://www.mathworks.com).

The failure probability of failure time *t* is an approximate straight line after logarithmic transformation. Figure 1 is the failure probability diagram of insulating oil at different high voltages under median rank estimate.

It can be seen from Fig. 1 that the slope of failure probability line at two stress levels of 26 kV and 32 kV is small, and the slope of the other five voltages are basically parallel. The main reason is that there are only 3 failure data obtained at the lowest stress level of 26 kV. The first failure datum 5.79 is particularly far less than the other two data 1579.52 and 2323.70, so the estimators have a certain deviation.

Akaike information criterion (AIC) and Bayesian information criterion (BIC)^36^ are most widely used in selecting the optimal model, the values of AIC and BIC are given by

38 $${\text{AIC}} = - 2\max \ln L + 2p,{\text{ BIC}} = - 2\max \ln L + p\ln q$$

where *p* is the number of estimated parameters, *q* is the number of all lifetime, and maxln*L* is the maximized log-likelihood. Table 3 shows the comparison results of model parameter estimation using MLE with the midpoint estimate and median rank estimate.

**Table 3:** Results comparison for model parameters with the different methods.

Method	*β*	*N*	*K*		Ln*L*(*β*, *N*, *K*)		AIC	BIC
			*K*_1_	*K*_2_	Ln*L*(*β*, *N*, *K*_1_)	Ln*L*(*β*, *N*, *K*_2_)		
MLE with midpoint estimate	0.8671	17.7311	1.65E−29	6.42E−29	− 338.5120	− 301.6495	609.2990	616.2912
MLE with median rank estimate	0.8017	17.7318	2.23E−29	6.70E−29	− 323.3626	− 300.8841	607.7682	614.7604
Results of Ref.^35^	0.7770	17.7296	6.87E−29		− 300.8174		607.6348	614.6270

From Table 3, it is found that the MLE with median rank is superior to MLE with midpoint estimate, the former has a larger log-likelihood value and smaller AIC and BIC values. Compared with the result of Ref.^35^, the relative errors of log-likelihood estimation, AIC and BIC are all within 0.022%, indicating that the method proposed in this paper has a high estimation accuracy. Therefore, the estimators of model parameter are given as follows: $\hat{\beta }$ = 0.8017, $\hat{N}$ = 17.7318 and $\hat{K}$ = 6.70E-29, respectively.

The variance–covariance matrix of model parameters *β*, *N*, *K* is given by:

$$\left[ {\begin{array}{*{20}c} {{\text{Var}}(\hat{\beta })} & {{\text{Cov}}(\hat{\beta },\hat{N})} & {{\text{Cov}}(\hat{\beta },\hat{K})} \\ {{\text{Cov}}(\hat{N},\hat{\beta })} & {{\text{Var}}(\hat{N})} & {{\text{Cov}}(\hat{N},\hat{K})} \\ {{\text{Cov}}(\hat{K},\hat{\beta })} & {{\text{Cov}}(\hat{K},\hat{N})} & {{\text{Var}}(\hat{K})} \\ \end{array} } \right] = \left[ {\begin{array}{*{20}c} {4.8882 \times 10^{ - 3} } & { - 5.1966 \times 10^{ - 4} } & { - 1.1305 \times 10^{ - 31} } \\ { - 5.1966 \times 10^{ - 4} } & {9.0867 \times 10^{ - 1} } & { - 2.1224 \times 10^{ - 28} } \\ { - 1.1305 \times 10^{ - 31} } & { - 2.1224 \times 10^{ - 28} } & {4.9679 \times 10^{ - 56} } \\ \end{array} } \right]$$

The normal operating voltage of insulating oil is *U*_0_ = 20 kV, therefore, using Eq. (28), the mean lifetime under normal use circumstances is 1.4406E + 05 min, the corresponding variance is Var(*t*_MTBF_) = 3.2824E + 08, based on Eqs. (25), (27) and (29), the interval estimator of mean lifetime is [1.2247E + 04, 4.9916E + 05]. The other point estimation and 95% confidence interval estimation of reliability indices and model parameters for transformer insulation oil under normal operating circumstances are shown in Table 4. Note that the values of reliability and failure rate are all given with mean lifetime 1.4406E + 05, reliable life is calculated by 90% reliability. Therefore, *u* = 9.8895E−02, *v* = 8.9476, *w* = − 12.0001, the corresponding variances are Var(*u*) = 1.5772E−01, Var(*v*) = 3.2893E−01 and Var(*w*) = 1.6033E−01, respectively.

**Table 4:** Reliability analysis results for insulating oil.

Parameter and reliability indices	Point estimator	Interval estimation with 95% confidence
*β*	0.8017	[0.6757, 0.9387]
*N*	17.7318	[15.9585, 19.6002]
*K*	6.70E−29	[9.76E−32, 5.04E−28]
*t*_MTBF_/min	1.4406E + 05	[1.2247E+04, 4.9916E+05]
*R*(*t* = *t*_MTBF_)	0.3316	[0.0903, 0.6024]
*t*(*R* = 0.9)/min	7689.68	[2673.85, 23,664.69]
*λ*(*t* = *t*_MTBF_)	6.1435E-06	[2.8027E-06, 1.3133E-05]

It can be seen from Table 4, the insulating oil has a higher mean lifetime 1.4406E + 05, but its reliability at mean lifetime is very low, only 33.16%, and the reliable life of insulating oil with 90% reliability is 7689.68 min, only 5.34% of mean lifetime. At this case, if the average life is taken as the criterion of maintenance and replacement only, it will bring some potential safety hazards. Therefore, the replacement time should be determined according to the reliability and mean lifetime of the transformer during maintenance simultaneously.

**Figure 2:**
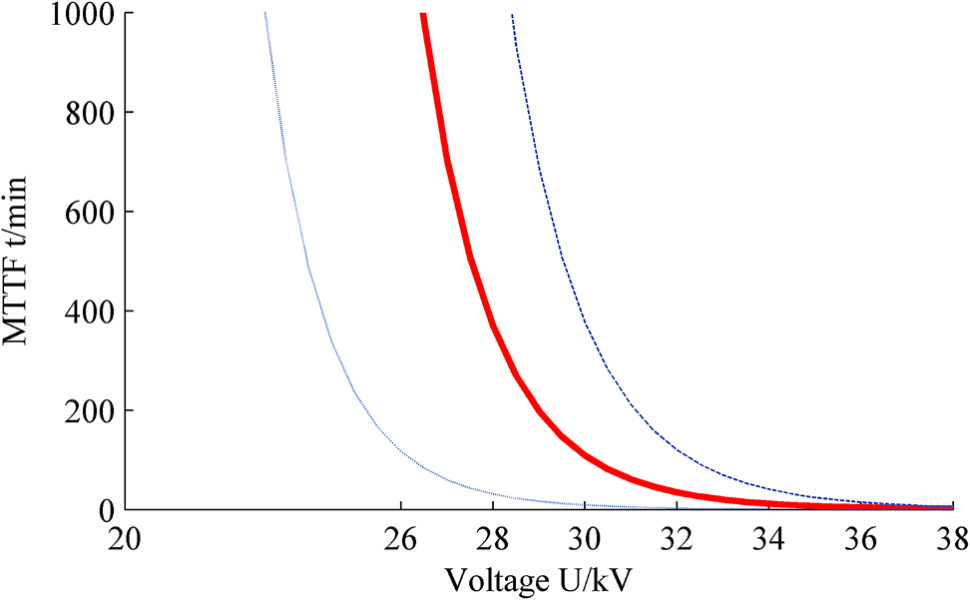
Interval estimation of MTTF. Figure created using Matlab R2014a (https://www.mathworks.com).

Figure 2 is the plot of mean time-to-failure vs. voltage, two sides confidence bounds are also given. It can be found that as the increasing of voltage, the MMTF decreases rapidly. The MMTFs are 1374.43 min and 369.34 min when voltages are 26 kV and 28 kV, especially when voltage is greater than 30 kV, the value of MMTF not exceeds 200 min. At 38 kV, it is 1.64 kV only.

**Figure 3:**
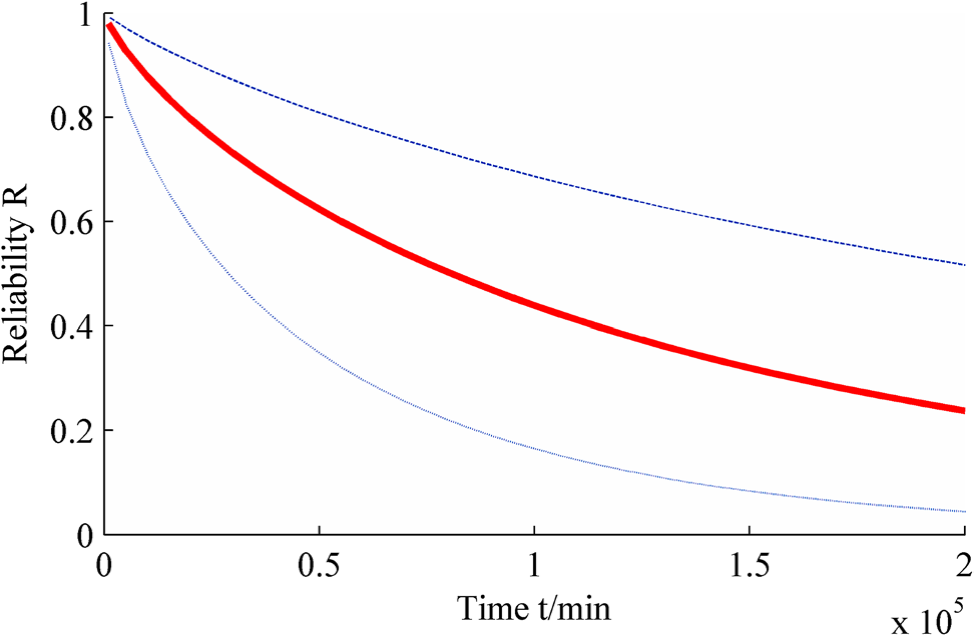
Interval estimation of reliability. Figure created using Matlab R2014a (https://www.mathworks.com).

**Figure 4:**
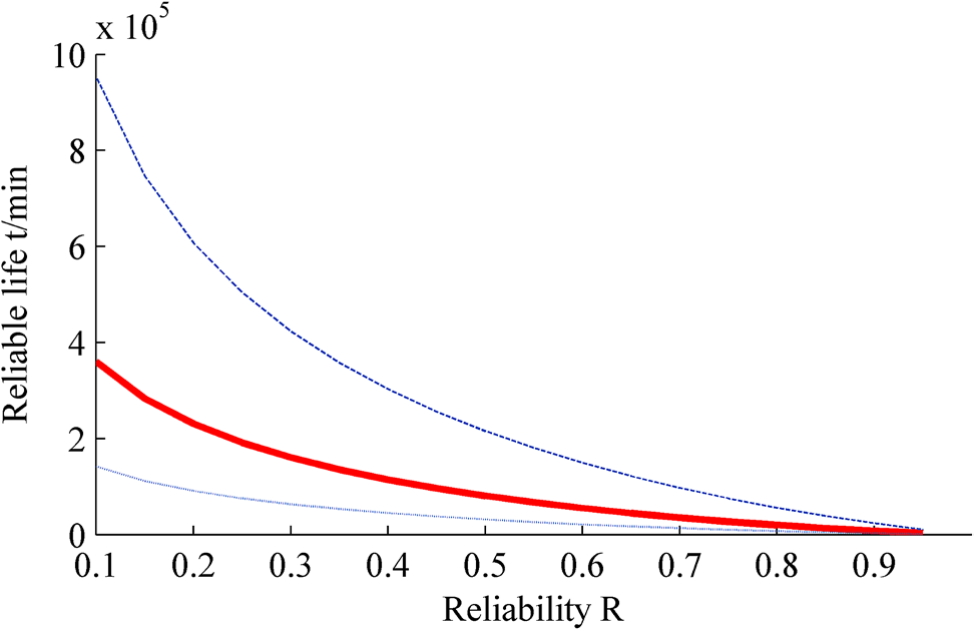
Interval estimation of reliable life. Figure created using Matlab R2014a (https://www.mathworks.com).

**Figure 5:**
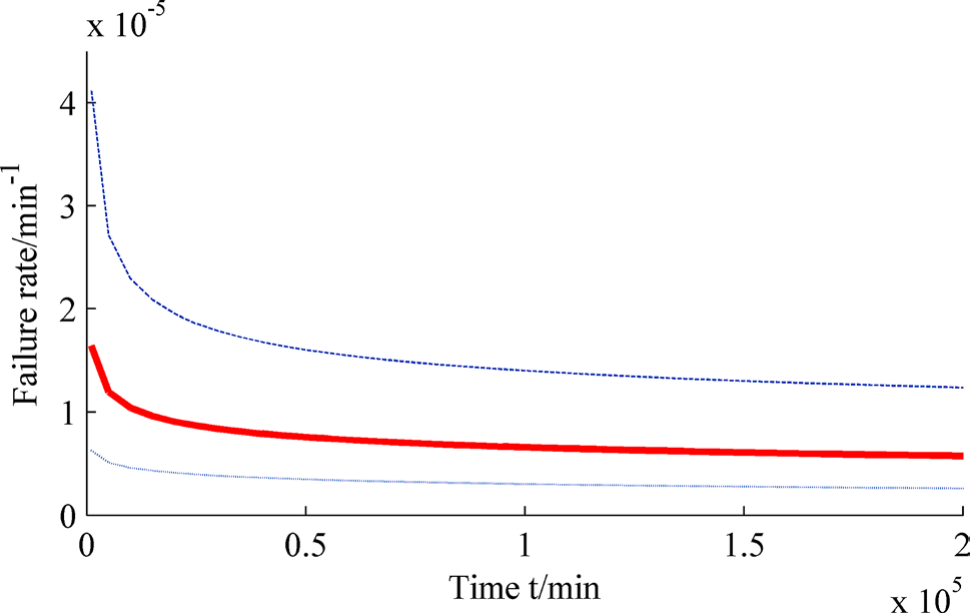
Interval estimation of failure rate. Figure created using Matlab R2014a (https://www.mathworks.com).

The interval estimations of reliability, reliable life and failure rate of insulating oil under normal use conditions are shown in Figs. 3, 4, 5.

## Conclusions

Based on the theory of constant ALT, a probabilistic method for reliability assessment of transformer insulating oil is proposed using two-parameter Weibull IPL life model. Numerical procedure of model parameter estimation is given. The reliability indices such as the MMTF, reliability, reliable life and failure rate of insulating oil under normal service conditions were predicted by using the proposed method, and the point estimation and interval estimation were calculated. Compared with the existing method, the method proposed in this paper has a high accuracy, the relative errors of log-likelihood estimation, AIC and BIC do not exceed 0.022%. The results of reliability analyses show that even the insulating oil has a higher mean lifetime, the reliable life is not always high. Therefore, mean lifetime and reliable life should be considered simultaneously in replacement and maintenance of insulating oil.

## Data Availability

All data generated or analysed during this study are included in this published article.
